# Evaluation of myotonometry for myotonia, muscle stiffness and elasticity in neuromuscular disorders

**DOI:** 10.1007/s00415-023-11867-z

**Published:** 2023-07-17

**Authors:** Katharina Lukas, Kristina Gutschmidt, Benedikt Schoser, Stephan Wenninger

**Affiliations:** Department of Neurology, Friedrich-Baur-Institute, LMU University Hospital, Ludwig-Maximilians University Munich, Ziemssenstr. 1, 80336 Munich, Germany

**Keywords:** Motoneuron disease, Myotonic dystrophies, Non-myotonic dystrophies, Myotonometry, Ultrasound imaging, Dynamometry

## Abstract

Neuromuscular disorders show extremely varied expressions of different symptoms and the involvement of muscles. Non-invasively, myotonia and muscle stiffness are challenging to measure objectively. Our study aims to test myotonia, elasticity, and stiffness in various neuromuscular diseases and to provide reference values for different neuromuscular disease groups using a novel handheld non-invasive myometer device MyotonPRO^®^. We conducted a monocentric blinded cross-sectional study in patients with a set of distinct neuromuscular diseases (NCT04411732, date of registration June 2, 2020). Fifty-two patients in five groups and 21 healthy subjects were enrolled. We evaluated motor function (6-min walk test, handheld dynamometry, Medical Research Council (MRC) Scale) and used ultrasound imaging to assess muscle tissue (Heckmatt scale). We measured muscle stiffness, frequency, decrement, creep, or relaxation using myotonometry with the device MyotonPRO^®^. Statistically, all values were calculated using the *t* test and Mann–Whitney *U* test. No differences were found in comparing the results of myotonometry between healthy and diseased probands. Furthermore, we did not find significant results in all five disease groups regarding myotonometry correlating with muscle strength or ultrasound imaging results. In summary, the myometer MyotonPRO^®^ could not identify significant differences between healthy individuals and neuromuscular patients in our patient collective. Additionally, this device could not distinguish between the five different groups of disorders displaying increased stiffness or decreased muscle tone due to muscle atrophy. In contrast, classic standard muscle tests could clearly decipher healthy controls and neuromuscular patients.

## Introduction

In neuromuscular diseases, overall, muscular involvement is highly heterogeneous. This is visible in the various neuromuscular disorders and within one type of disease itself. Due to the varied manifestations of neuromuscular disease among patients, diagnosis is often tricky, frequently leading to a prolonged period between symptom onset and diagnosis [1]. Myotonia, stiffness, muscle atrophy, and weakness are key symptoms of myotonic dystrophies [2]. For non-dystrophic myotonia, generalised muscle stiffness is the most common symptom [3]. Additionally, muscles are selectively affected by fatty infiltration and atrophy [4]. In patients suffering from motoneuron disease, symptoms range from atrophy to severe spasticity and stiffness [5]. Other than non-myotonic diseases, motoneuron disease as well as demyelinating polyneuropathy (CIDP) or different forms of hereditary sensory and motor neuropathies (HMSN) typically do not show fatty infiltration in the affected muscle tissue [6, 7]. Because detection of delayed relaxation and increased stiffness after a voluntary contraction is possible in both dystrophic and non-dystrophic myotonia [3], as well as motoneuron disease, and because of the wide variety in phenotypes of all conditions, clinical diagnosis stays challenging.

Non-invasively, myotonia and muscle stiffness are difficult to measure objectively but are typically only described clinically. Semi-invasively, the severity of myotonia is best assessed by electromyography. This requires a high logistic effort and technical equipment, including sophisticated computerised measurements and a trained neurophysiologist, to be readily available. This implementation in routine clinical practice is almost impossible. Ultrasound of muscle tissue has proven to be an excellent tool in detecting neuromuscular disease. It has shown high sensitivity and specificity for characterising various diseases but is mostly complementary to electrophysiological or clinical results [8].

To better understand and assess muscle stiffness and myotonia, the current study used a handheld myometer device that measures muscle fibres' elasticity, stiffness, and relaxation time non-invasively. In this study, we used the commercially available device MyotonPro^®^, which is used to evaluate the parameters previously mentioned.

Its validity and reliability were already proven in a large cohort of healthy volunteers and patients with different types of central nervous system diseases affecting the muscles, such as stroke or Parkinson’s disease. Assessing patients with stroke, the device can be a reliable source determining pre- and posttreatment abilities [9]. Concerning patients with Parkinson’s disease, literature shows a significant difference in rigidity and stiffness values between healthy individuals and patients with Parkinson’s disease, seeing a higher rigidity and correlating higher value for stiffness in myotonometry [10]. While finding significant differences in patients with or without paratonia concerning the MyotonPRO^®^ values, careful interpretation of results concerning patients with paratonia is necessary [11]. One study aimed to inspect minimal detectable changes in muscles and compare them to ultrasound imaging, which showed significant results for both measurements, determining that myotonometry can detect minimal changes in muscles [12].

The application of short pulses of the MyotonPRO^®^ to the underlying tissue will trigger a muscle deformation, and the resulting oscillation will be measured and analysed by the device [13]. We hypothesised that this device might allow a statement concerning the severity of myotonia or stiffness and could be used in evaluating disease progression. So far, the knowledge of muscle elasticity and stiffness in various neuromuscular diseases is scarce.

Our study aims to test elasticity, myotonia and stiffness in various neuromuscular diseases by using the MyotonPRO^®^ and to investigate whether there is clinical applicability in the follow-up and treatment of neuromuscular disorders.

## Methods

### Study setting and patient population

We conducted a monocentric blinded cross-sectional study in patients with neuromuscular diseases to assess stiffness, muscle tone, relaxation periods and elasticity of various muscles. Our secondary objective was to provide reference values for the measured entities of multiple muscles in patients with myotonic or non-myotonic neuromuscular disorders and to assess correlations to compare result values with clinical muscle function tests measured by clinical evaluation (MRC-scale) and the 6-min walk test, and to assess correlations between subcutaneous fat, muscle thickness and echogenicity.

Inclusion criteria consisted of willingness to provide signed informed consent and being able to perform muscular function tests and the non-invasive measuring with the MyotonPRO^®^ device. Only adult patients ≥ 18 years of age with neuromuscular disease were included. After providing informed consent, eligible patients were included and underwent several conventional muscle tests. Subsequently, we measured with the MyotonPRO^®^ device on two separate occasions to rule out gross measurement errors. Patients were monitored continuously during the implementation of the measurement so possible side effects would be detected. Exclusion criteria included severe comorbidities, inability to perform required muscle function tests or, according to the investigator, inability to adhere to the specifications and requirements of the study. Additionally, participating in another clinical study using investigational treatment was an exclusion criterion.

Fifty-two patients were enrolled and divided into five disease groups. Additionally, 21 healthy subjects were enrolled as a control group. All included patients were assessed at the neuromuscular centre Friedrich-Baur-Institute at the Ludwig-Maximilians University Munich, Germany. The study was approved by the ethics committee of the LMU university hospital, Project No. 19-613, and the protocol was registered on a public clinical trials registry (ClinicalTrials.gov Identifier NCT04411732).

### Examination and methods

First, all eligible patients were categorised into one of the five following groups: motoneuron disease (e.g. amyotrophic lateral sclerosis, spinal muscular atrophy), non-myotonic myopathy (e.g., central core disease, Pompe disease), myotonic myopathy (e.g. myotonic dystrophy type 1 or 2), peripheral neuropathy (e.g. critical illness polyneuropathy, multifocal motor neuropathy), and myositis (e.g. polymyositis, necrotic myositis).

Upon first examination, muscle strength was measured using the MRC (Medical Research Council) scale. MRC score ranged from 5 (maximum muscle force) to 0 (no movement detectable) [14]. Additionally, we used dynamometry to assess the maximum applied pressure in kg in all measured muscles: thenar and hypothenar, m. biceps brachii, m. triceps brachii, m. deltoideus, m. quadriceps femoris, m. tibialis anterior, m. gastrocnemius on both sides, respectively. We used the best three attempts to maximise precise results [15].

To assess general muscle function, we conducted the 6-min walk test (6MWT). Subjects were asked to walk on a hard, flat surface as fast as they could, and the overall distance was measured [16]. Standard values for the 6MWT are a walking distance greater than 637 m in healthy adults [17]. In addition, we conducted a standardised muscle ultrasound to determine the diameter of muscle mass, subcutaneous fat, and underlying tissue. Muscle ultrasound is an ideal imaging modality for non-invasive, atraumatic, and radiation-free neuromuscular imaging.

We also documented muscle intensity using the four-point-Heckmatt scale [18]. To better understand pain related to the presented disorders, we also collected data using the Numeric Rating Scale (NRS) [19]. Patients were asked to rate their pain level using the NRS in the muscles, as mentioned above.

For measuring muscle stiffness, elasticity and relaxation time, the commercially available device MyotonPRO^®^ was used. MyotonPRO^®^ is a non-invasive, hand-held device placed perpendicular to the underlying muscle tissue. Using little pressure, the device gets activated. A light signal will indicate if the probe is pushed down adequately and if the pre-compression load is met. Using a brief mechanical impulse, the device then exerts a local impact on the examined muscle. This results in a slight muscle deformation. The damped oscillatory behaviour of the underlying tissue will be recorded after the stimulation, and viscoelastic stiffness will be calculated automatically. The following measurements will be performed automatically, analysing the oscillation curves, and calculating the tissue tone [frequency (Hz), the tissue stiffness (N/m)], the decrement as a parameter for the elastic stiffness of the tissue and viscoelastic parameters like relaxation time [in milliseconds (ms)] and creep as non-elastic tissue strain, also known as retardation [20]. The device will automatically calculate the coefficient of variation (CV) between sets, using sets of five impulses in this study. After the measurement is completed, the coefficient of variation will be displayed as a percentage next to every parameter [12]. The functionality of the MyotonPRO^®^, measurement and calculations have been tested and validated in many clinical studies [9, 10, 13]. Therefore, this study is not intended to validate the measurements of the MyotonPRO^®^ device itself nor its safety or accuracy of measures. The primary outcome is to evaluate measurement results in patients with neuromuscular diseases and elaborate reference values. The muscles examined with the MyotonPRO^®^ were Mm. deltoideus, biceps brachii, triceps brachii, opponens pollicis, abductor digiti minimi, rectus femoris, tibialis anterior, gastrocnemius caput mediale (both sides each).

### Statistical analysis

Microsoft Excel^®^ version 16 and SPSS Statistics^®^ version 29 were used to analyse the presented data. All data were checked for normal distribution using the Kolmogorov–Smirnov-Test. The significance level (alpha) was set at ≤ 0.05. Next, we performed an unpaired, two-sided *t* test for all metric, normally distributed values. For all non-parametric values, we performed the Mann–Whitney *U* test. For purposes of clarity, only the results of the biceps brachii muscle are presented in this paper. All other muscles examined yielded comparable results in the evaluation and are available on reasonable request.

## Results

### Baseline demographic and characteristics

Characteristics of the study population are presented in Table 1. Patients had a mean age of 48.7 years, while the control subjects were 50.1 years old on average. Female gender was found in 40.4% of patients and 47.6% in the healthy group. No statistically significant differences were found between men and women regarding baseline demographics, reflecting normally distributed values. Regarding the MRC scale, a significantly lower overall sum could be detected in neuromuscular patients compared to healthy individuals (*p* < 0.001), linking to an overall lower muscle force in patients. In all five different disease groups, there was no statistically significant difference in gender or age, apart from a significant difference in age in patients with myotonic myopathies versus healthy subjects (*p* = 0.012).

**Table 1:** Characteristics of study populations

	Healthy subjects *n* = 21 (%)	Diseased subjects *n* = 52 (%)	*p* value
Age, years (mean ± SD)	50.1 ± 22	48.7 ± 17	< 0.80
Female gender	10 (47.6%)	21 (40.4%)	0.57 *χ*^2^ test
Height^a^ (mean ± SD)	1.74 ± 0.07	1.75 ± 0.1	< 0.70
Weight^b^ (mean ± SD)	71.5 ± 15.0	75.6 ± 19.5	< 0.38
Handedness (right-handed)	16 (76.2%)	48 (92.3%)	**0.03** **Fisher-test**
MRC^c^ (score sum) (mean ± SD)	79.6 ± 0.8	71.2 ± 9.8	** < 0.001**

Significant results (*p* < 0.05) are highlighted. Unless otherwise stated, the *t* test was performed

^a^Measured in meters

^b^Measured in kilogram

^c^MRC = medical research council, taxonomy standard to measure muscle strength by resisted isometrics on a scale from 0 to 5

### Conventional muscle testing

All data are presented in Table 2. In the conducted 6MWT, five (1%) patients could not perform the test due to being unable to walk more than a few steps or being wheelchair dependent. Standard values for the 6MWT are a walking distance greater than 637 m (m) in healthy adults. The enrolled patients showed a significantly lower mean distance of 431 m (*p* < 0.001), only reaching 67% of healthy subjects. Regarding the muscle tissue ultrasound imaging, we classified the muscle tissue using the four-point Heckmatt scale. Muscle is being categorised in one of the following grades: grade 1: normal muscle echogenicity; grade 2: increased muscle echogenicity with regular bone reflection; grade 3: increased muscle echogenicity with reduced bone reflection; grade 4: markedly increased muscle echogenicity with loss of bone reflection [21]. Healthy subjects had a grade 1, which classifies healthy muscle tissue, in 95.2% of all cases. On the other hand, neuromuscular patients are classified as grade 1 in 56.1% of all patients, which is significantly lower than the control group (*p* = 0.006).

**Table 2:** Standard muscle tests, using the example of the biceps brachii muscle

	Healthy subjects *n* = 21 (%)	Neuromuscular patients *n* = 52 (%)	*p* value
6MWT^a^ (mean ± SD)	660.8 ± 68.2	431.1 ± 171.3	** < 0.001**
Heckmatt scale < 2^b^	20 (95.2%)	23 (56.1%)^c^	**0.006** **Fisher-Test**

Significant results (*p* < 0.05) are highlighted. Unless otherwise stated, the *t* test was performed

^a^Measured in the distance walked in meters

^b^Heckmatt scales: grade 1: normal muscle echogenicity; grade 2: increased muscle echogenicity with normal bone reflection; grade 3: increased muscle echogenicity with reduced bone reflection; grade 4: markedly increased muscle echogenicity with loss of bone reflection [21]

^c^*N* = 41 subjects

In the recorded results of the NRS regarding pain, the mean result was a pain level of 1 among the patient group.

### Objective assessments: dynamometry and MyotonPRO^®^ results

**Fig. 1:**
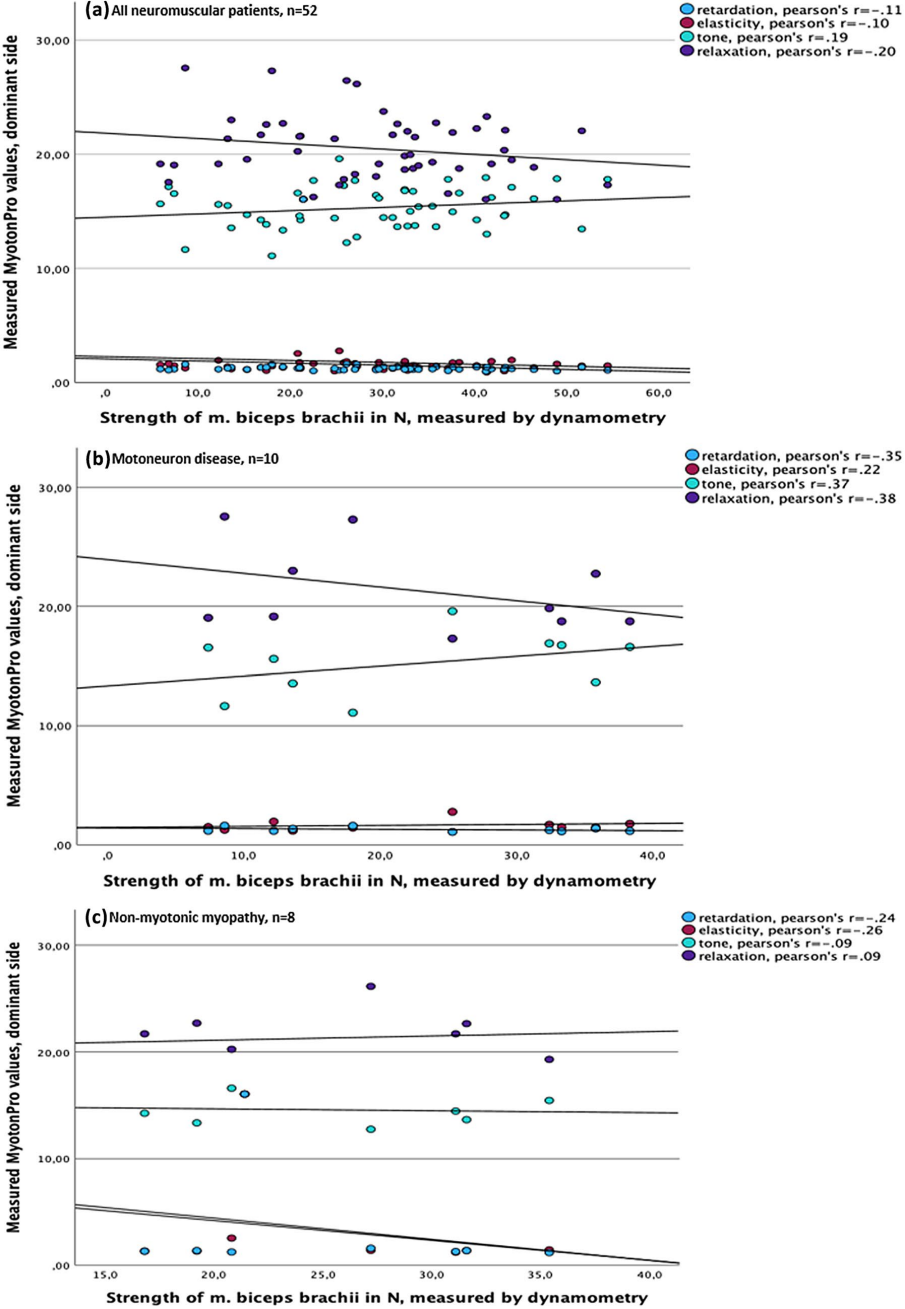

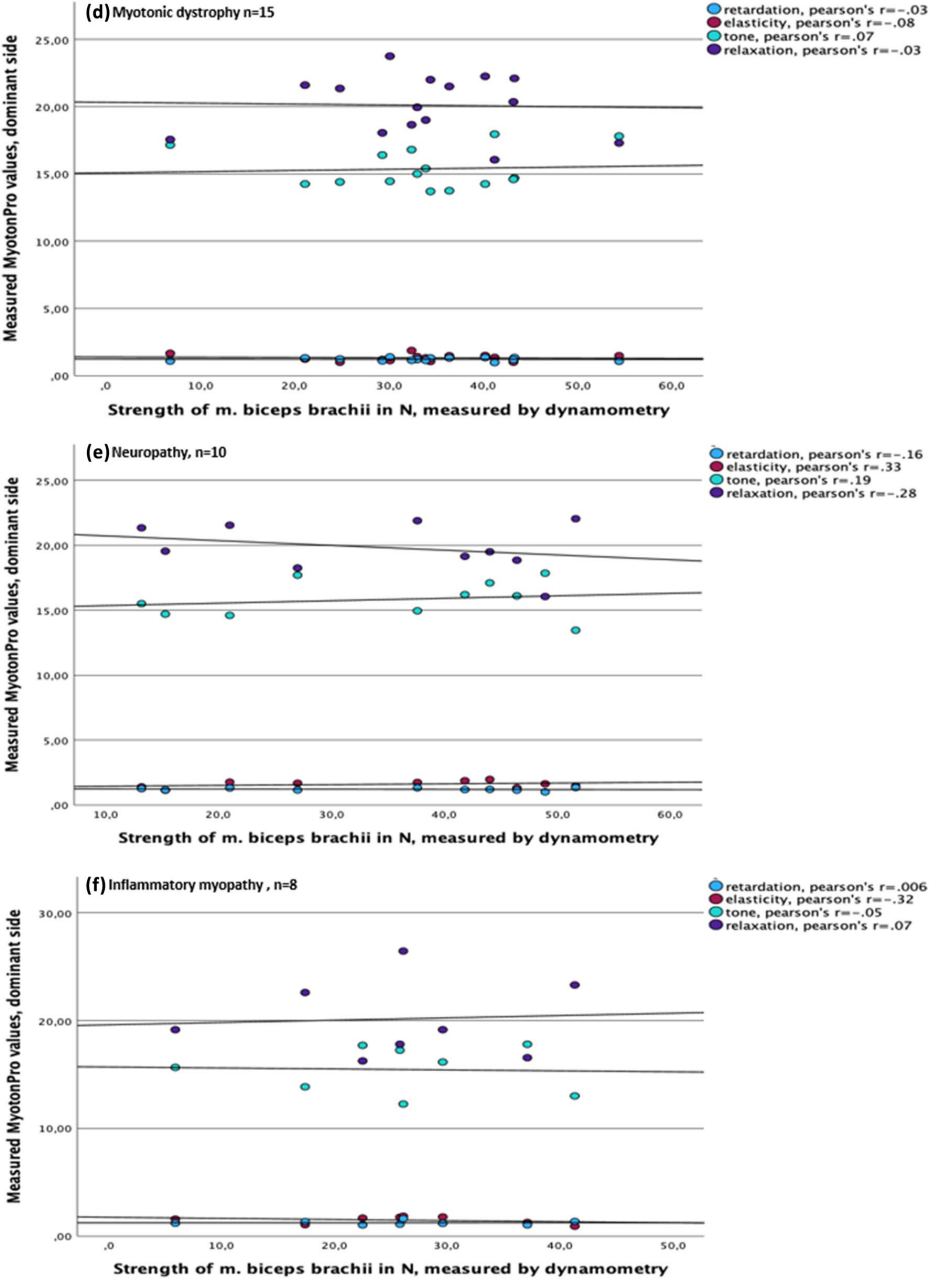
Correlation between MyotonPro^®^ values^1^ and strength of the biceps brachii muscle on the dominant side. ^1^Stiffness is not depicted due to clarity reasons

The correlation between parameters of the MyotonPRO^®^, such as frequency, decrement, relaxation and creep, and the measured muscle force using the example of the bicep brachii muscle of the dominant side is presented in Fig. 1. In the examined muscle, no correlation could be detected between the values of the MyotonPRO^®^ and muscle strength. In the conducted sub-analyses between all patient groups, it was impossible to establish a correlation between changed muscle properties and the measurement result. Pearson’s *r* for all measurements was < 0.5, showing no significant correlation. In some cases, Pearson’s *r* even showed negative figures, but also not significantly. Due to clarity reasons, high stiffness results could not be depicted in the graph, but also offered a low Pearson’s *r* for all defined groups and overall, resulting in no correlation between muscle force and stiffness.

### Parameters of the MyotonPRO^®^

Table 3 compares the healthy control group and neuromuscular patients regarding the values of the MyotonPRO^®^ with the example of the bicep brachii muscle. No significant differences between healthy and diseased probands were detected in all neuromuscular patients. For the different disease groups, all results are presented in Tables 4, 5, 6, 7 and 8. There were significant results in non-myotonic myopathies such as Pompe or central core disease. The patients showed a significantly lower muscle tone (*p* = 0.017), stiffness (*p* = 0.021) and creep (retardation, *p* < 0.001) compared to healthy subjects. No significant differences between healthy probands and neuromuscular patients could be detected in all other groups. Depicted in Table 9 are results for non-myotonic myopathies in the rectus femoris muscle, where no significant differences could be found. Like the presented data, other muscles, such as the rectus femoris muscle, did not show significant differences between healthy and sick probands.

**Table 3:** Statistical comparison of MyotonPro^®^ measured values between neuromuscular patients and the control group using the example of the biceps brachii muscle

Biceps brachii muscle	Dominant side^a^		
Categories	Control group *n* = 21	Neuromuscular patients *n* = 52	*p* value^b^
Tone (mean ± SD)	15.67 ± 1.02	15.34 ± 1.81	0.33
Stiffness (mean ± SD)	263.721 ± 28.46	252.43 ± 51.63	0.26
Elasticity (median, min–max)	1.37 (0.88–2.42)	1.45 (0.91–16.05)	0.35^c^
Relaxation (mean ± SD)	19.64 ± 1.82	20.44 ± 2.80	0.15
Retardation (median, min–max)	1.18 (0.99–1.43)	1.21 (0.98–16.05)	0.17^c^

^a^Dominant side was considered, healthy subjects = 5 left-handed, patients with motoneuron disease = 2 left-handed

^b^Significant results (*p* < 0.05) are highlighted. *T* test was performed unless otherwise indicated

^c^Mann–Whitney *U* test was performed

**Table 4:** Statistical comparison of MyotonPro^®^ measurements between motoneuron disease patients and the control group using the example of the biceps brachii muscle

Biceps brachii muscle	Dominant side^a^		
Categories	Control group *n* = 21	Motoneuron patients *n* = 10	*p* value^b^
Tone (mean ± SD)	15.96 ± 1.30	15.19 ± 2.65	0.41
Stiffness (median, min–max)	261.5 (204–338.5)	272 (183–297.5)	0.57^c^
Elasticity (mean ± SD)	1.41 ± 0.33	1.65 ± 0.45	0.15
Relaxation (mean ± SD)	19.24 ± 1.96	21.25 ± 3.66	0.12
Retardation (mean ± SD)	1.17 ± 0.11	1.29 ± 0.19	0.09

^a^Dominant side was considered, healthy subjects = 5 left-handed, patients with motoneuron disease = 0 left-handed

^b^Significant results (*p* < 0.05) are highlighted. *T* test was performed unless otherwise indicated

^c^Mann–Whitney *U* test was performed

**Table 5:** Statistical comparison of MyotonPro^®^ measurements between patients with non-myotonic myopathies and the control group using the example of the biceps brachii muscle

Biceps brachii muscle	Dominant side^a^		
Categories	Control group n = 21	Patients with non-myotonic myopathies n = 8	*p* value^b^
Tone (mean ± SD)	15.96 ± 1.30	14.57 ± 1.35	**0.017**
Stiffness (median, min–max)	261.5 (204–338.5)	229.8 (16–277.5)	**0.021**^c^
Elasticity (median, min–max)	1.30 (0.88–2.24)	1.40 (1.23–16.05)	0.30^a^
Relaxation (mean ± SD)	19.24 ± 1.96	21.31 ± 2.93	0.036
Retardation (median, min–max)	1.15 (0.99–1.43)	1.34 (1.19–16.05)	** < 0.001**^c^

^a^Dominant side was considered, healthy subjects = 5 left-handed, patients with myotonic dystrophy = 0 left-handed

^b^Significant results (*p* < 0.05) are highlighted. *T* test was performed unless otherwise indicated

^c^Mann–Whitney *U* test was performed

**Table 6:** Statistical comparison MyotonPro® of measured values between patients with myotonic myopathy and the control group using the example of the biceps brachii muscle

Biceps brachii muscle	Dominant side^a^		
Categories	Control group *n* = 21	Patients with myotonia *n* = 16	*p* value^b^
Tone (mean ± SD)	15.67 ± 1.02	15.45 ± 1.44	0.61
Stiffness (mean ± SD)	263.21 ± 28.46	257.07 ± 36.16	0.57
Elasticity (mean ± SD)	1.47 ± 0.42	1.33 ± 0.23	0.23
Relaxation (mean ± SD)	19.64 ± 1.82	19.97 ± 2.22	0.62
Retardation (mean ± SD)	1.19 ± 0.11	1.21 ± 0.12	0.74

^a^Dominant side was considered, healthy subjects = 5 left-handed, patients with myotonia = 2 left-handed

^b^Significant results (*p* < 0.05) are highlighted. *T* test was performed unless otherwise indicated

**Table 7:** Statistical comparison MyotonPro^®^ of measured values between patients with neuropathy and the control group using the example of the biceps brachii muscle

Biceps brachii muscle	Dominant side^a^		
Categories	Control group *n* = 21	Patients with neuropathy *n* = 10	*p* value^b^
Tone (mean ± SD)	15.96 ± 1.30	15.82 ± 1.44	0.78
Stiffness (mean ± SD)	268.79 ± 35.11	266.10 ± 34.35	0.84
Elasticity (mean ± SD)	1.41 ± 0.33	1.59 ± 0.26	0.12
Relaxation (mean ± SD)	19.24 ± 1.96	19.82 ± 1.91	0.45
Retardation (mean ± SD)	1.17 ± 0.11	1.21 ± 0.10	0.39

^a^Dominant side was considered, healthy subjects = 5 left-handed, patients with neuropathy = 0 left-handed

^b^Significant results (*p* < 0.05) are highlighted. *T* test was performed unless otherwise indicated

**Table 8:** Statistical comparison MyotonPro^®^ of measured values between patients with myositis and the control group using the example of the biceps brachii muscle

Biceps brachii muscle	Dominant side^a^		
Categories	Control group *n* = 21	Patients with neuropathy *n* = 8	*p* value^b^
Tone (mean ± SD)	15.96 ± 1.30	15.46 ± 2.18	0.56
Stiffness (mean ± SD)	268.79 ± 35.11	271.06 ± 56.97	0.92
Elasticity (mean ± SD)	1.41 ± 0.33	1.47 ± 0.35	0.62
Relaxation (mean ± SD)	19.24 ± 1.96	20.16 ± 3.61	0.52
Retardation (mean ± SD)	1.17 ± 0.11	1.23 ± 0.20	0.33

^a^Dominant side was considered, healthy subjects = 5 left-handed, patients with myositis = 0 left-handed

^b^Significant results (*p* < 0.05) are highlighted. *T* test was performed unless otherwise indicated

**Table 9:** Statistical comparison of MyotonPro^®^ measurements between patients with non-myotonic myopathies and the control group using the example of the rectus femoris muscle

Rectus femoris muscle	Dominant side^a^		
Categories	Healthy control group *n* = 21	Patients with non-myotonic myopathy *n* = 8	*p* value^b^
Tone (mean ± SD)	16.28 ± 3.14	15.68 ± 2.78	0.64
Stiffness (mean ± SD)	294.0 ± 74.61	255.21 ± 108.60	0.28
Elasticity (median, min–max)	1.61 (0.87–2.81)	1.92 (1.48–18.7)	0.07^c^
Relaxation (mean ± SD)	19.67 ± 4.09	20.70 ± 3.81	0.54
Retardation (median, min–max)	1.31 (0.71–1.53)	1.38 (1.04–18.7)	0.24^c^

^a^Dominant side was considered, healthy subjects = 5 left-handed, patients with motoneuron disease = 0 left-handed

^b^Significant results (*p* < 0.05) are highlighted. *T* test was performed unless otherwise indicated

^c^Mann–Whitney-test was performed

### Study limitations

In our patient cohort, only a small number of patients were enrolled in each group, making a comprehensive statistical analysis of factors influencing stiffness and elasticity not reasonable. On the other hand, this is the first study in a sufficient number of patients with different neuromuscular disorders that were assessed using hand-held myotonometry and comparing the measured values with conventional muscle testing and clinical assessments. The lack of participant characteristics such as subcutaneous fat thickness, % body fat, body mass index and activity level can be seen as a further limitations of the study. However, correlations to the standard clinical assessments such as muscle strength, 6MWT and muscle ultrasound have been performed.

## Discussion

We conducted a pilot study to examine the values of hand-held myotonometry in neuromuscular patients in classified disease groups and whether the device would be able to differentiate between the groups. Additionally, we wanted to understand whether this myotonometric device could be used in a clinical setting to deliver valuable input into a patient’s disease process and treatment. The hand-held device could not distinguish between healthy probands and neuromuscular patients in our patient cohort. As presented, all sub-groups, having different symptoms and expressions of stiffness, elasticity and myotonia, as well as the collective of neuromuscular patients, could not be differentiated using the MyotonPro^®^. No correlation was found regarding the comparison and correlation between dynamometry and myotonometry. For example, we hypothesised that we could decipher a correlation between high muscle force and a measured muscle tone. This was not the case in our analysis. The limited significant results, such as our results in the non-myotonic myopathies group, only depicted a small number of patients and were only found in a few selected muscles. Therefore, the applicability and sensitivity of measuring clinically meaningful changes using MyotonPRO^®^ are debatable. Overall, conventional muscle tests, such as the MRC scale, the 6-min-walk test, and ultrasound imaging with the Heckmatt scale classification, showed significantly reliable results and could identify differences. Myotonometry by MyotonPRO^®^ could not reliably predict measured values or generate hints about the underlying muscle disease.

## Data Availability

The data that support the findings of this study are available from the corresponding author upon reasonable request.
